# Impact of temperature, inoculum flow pattern, inoculum type, and their ratio on dry anaerobic digestion for biogas production

**DOI:** 10.1038/s41598-022-10025-1

**Published:** 2022-04-13

**Authors:** Md Shahadat Hossain, Tahmid ul Karim, Mahade Hassan Onik, Deepak Kumar, Md Anisur Rahman, Abu Yousuf, Mohammad Rakib Uddin

**Affiliations:** 1 Department of Chemical Engineering, State University of New York College of Environmental Science and Forestry, Syracuse, NY 13210 USA; 2 Department of Chemical Engineering & Polymer Science, Shahjalal University of Science and Technology, Sylhet, 3114 Bangladesh; 3 Department of Chemical Engineering, University of Massachusetts Amherst, Amherst, MA USA; 4 Dipartimento di Ingegneria, Università degli studi di Napoli “Parthenope”, 80143 Naples, Italy

**Keywords:** Biological techniques, Biotechnology, Chemical biology, Environmental sciences

## Abstract

This study is aimed to apply dry anaerobic digestion (DAD) for methane (CH_4_) enriched biogas production from unsorted organic municipal solid waste (MSW). Cumulative biogas production was monitored for 35 days of operation in batch digesters at fixed feedstock to inoculum (F/I) ratio 2. Anaerobic sludge (AS) and cow manure (CM) were used as inoculum in single and mixed modes. Several process parameters such as inoculum flow pattern (single layer, multilayer, and spiral), digestion temperature (25 to 40 °C), inoculation modes (single and mixed mode), and inoculation proportion (AS:CM = 1:1, 1:2, 1:3, and 2:1) were investigated to determine the optimum DAD conditions to maximize the CH_4_ laden biogas yield. The study of inoculum flow pattern showed that digester with multilayer inoculum configuration generated the maximum 555 mL cumulative biogas with the production rate of 195 mL/day (at 25 °C). Biogas production rate and cumulative biogas production were found to increase with a rise in temperature and the maximum values of 380 mL/day and 1515 mL respectively were observed at 37 °C. The mixed mode of inoculation containing AS and CM augmented the biogas yield at previously optimized conditions. Final results showed that digester with multilayer inoculum flow pattern at 37 °C produced 1850 mL cumulative biogas with 1256.58 mL CH_4_/kg volatile solid (VS) when the mixed inoculum was used at the AS:CM—1:2 ratio. Biogas production with this significant amount of CH_4_ justifies the use of the DAD process for energy (biogas) generation from widely available biomass feedstock (MSW), offering various advantages to the environment.

## Introduction

Fossil fuel reserves are declining continuously due to diversified use in the transportation and industrial sector. Consequently, there will be a severe energy crisis within the next few decades due to the rapid urbanization and industrialization around the globe. At the same time, the greenhouse gas (GHG) emissions from the production and use of fossil fuels raise several environmental concerns^1^. These energy crises and environmental concerns necessitate to invest in sustainable sources of renewable fuels. Municipal solid waste (MSW) is one such potential and abundantly available energy source^2,3^.

MSW management has become one of the major environmental problems faced by municipalities across the world. Rapid urbanization and rising standards of living are leading to higher amounts of solid waste generation globally, which is estimated to be around 1012 million metric tons by 2025^4^. Biogas production from the MSW through anaerobic digestion (AD) can be a sustainable solution to tackle both the MSW management and energy scarcity problems. Biogas production from biomass has experienced a 90% increase over the last decade (from 65 GW in 2010 to 120 GW in 2019) mostly due to increased concern about climate change, affordable price, and enhanced distribution networks^5^. In 2017, world biogas production was 58.7 billion Nm^3^ with a growth rate of 11.2%^6^. Only Europe contributed 70% of the total biogas generated at that time, with a biogas-based electricity generation of 64 TWh^5^. In total, more than 17,240 AD facilities were actively producing biogas in Europe during 2014^6^. In the USA, about 2000 AD plants were operating to manage the generated waste biomass in 2015 according to American Biogas Council^7^. At present, the potential for biomethane production is over 700 million tonnes of oil equivalent (Mtoe), whereas the current worldwide biomethane production is only about 3.5 Mtoe^5^.

AD process involves a series of microbial steps—hydrolysis, acidogenesis, acetogenesis, and methanogenesis—in which microorganisms break down organic biomass into renewable energy products (biogas), in absence of oxygen. Depending on the total solid (TS) in the slurry during the process, the AD can be categorized as wet anaerobic digestion (WAD) and dry anaerobic digestion (DAD). To utilize the biomethane potential and improve the biogas/biomethane yield, the DAD process has been studied extensively by varying feedstock, inoculum, and different process parameters. Choi et al*.*^8^ performed the DAD of food waste using sludge mixtures (dewatered sludge cake and mesophilic anaerobic sludge at 1:1 ratio) as inoculum under mesophilic conditions (37 °C). Over the 40 days of operation, 66% volatile solid (VS) reduction was observed, with a methane production rate of 2.51 m^3^/(m^3^ digester/day)^8^. DAD of corn stover (with/without NaOH pretreatment) in the presence/absence of chicken manure was conducted by Li et al*.*^9^ under mesophilic (37 °C) and thermophilic (50 °C) conditions^9^. The inoculum used was residual sludge from a biogas plant treating MSW. At an optimized F/I ratio of 3, the authors observed a 29.4 and 40.1% increase in biogas (386.3 mL/g of VS) and methane (194.8 mL/g of VS) yield for treated corn stover compared to those of untreated corn stover under thermophilic conditions. Under the mesophilic condition, the system failed due to the accumulation of volatile fatty acids (VFAs). Matheri et al.^10^ studied the biogas production from the organic fraction of MSW through the DAD process using cow manure as inoculum under the mesophilic optimum temperature of 37 °C at pH 7.0. The authors reported an absence of lag phase due to the presence of a balanced and active microbial community. Moreover, a higher methane production was observed under shorter hydraulic retention time owing to higher rate of biodegradation. Wang et al*.*^11^ used distilled grain waste as feed to produce biogas through the DAD process operating at thermophilic conditions (52 °C). The methane production was reported 139.4 mL/g to 190.5 mL/g VS. In another study on DAD of agricultural waste (mainly rice straw) with pig urine and dung (inoculum), a mean biogas yield of 580 ± 36 Nm^3^/VS was observed at a total solid (TS) loading of 27% and a retention time of 20 days under thermophilic conditions (55 °C)^12^.

Although the DAD processes provide an advantage of lower water use and smaller digester volume due to higher organic loading, it suffers from several limitations also. In the case of an inffective process operation, the DAD process can potentially release GHGs to the environment^14^. Generally, a long HRT is required for effective mass transfer and stabilization of microbial community within the digester. A post-treatment, such as hydrothermal carbonization of the digestate might also be necessary to lower the BOD, COD, and temperature within the acceptable limit before using it for soil amendment or releasing it to the environment. Poor startup performance, incomplete mixing, and accumulation of VFAs are considered as other limitations of the DAD process^13,15,16^. To overcome these difficulties and to improve efficiency of the DAD process, different kinds of bioreactors have been developed (summarized in Supplementary Table S1). However, the low methane fraction in the biogas restricts the commercial implementation of these systems. To enhance the methane percentage and develop a feasible DAD process, it is important to review the optimization techniques and suggest possible areas where improvements could be made. This may include optimization of digestion conditions like temperature, pH, buffering capacity, and VFA’s concentration. Other parameters also need to study such as digester configuration, solid retention time (SRT), feedstock type, organic loading rate (OLR), inoculum type, and co-digestion.

Therefore, the main objective of this study was to optimize the DAD process parameters for enhanced biogas production from the digestion of MSW by activated sludge (AS) and cow manure (CM) inoculum (in single and mixed-mode). As a part of optimization, digester configurations were also varied, the first objective was to investigate various inoculum flow patterns (single layer, multilayer, and spiral) to reduce the SRT. The second objective of the study was to determine a suitable digestion temperature at thermophilic range for higher biogas yield. To improve the biogas yield, a single and mixed-mode of AS and CM inoculation to the biomass (MSW) were also studied. Finally, different inoculum proportion at the mixed mode of inoculation was assessed to find out the optimum mixed inoculum ratio. The novelty of this study can be summarized as : (i) development of a process for unsorted (both degradable and non-degradable) MSW digestion (ii) systematic evaluation of the different digester configurations to lower the SRT for increased biogas yield and (iii) engineering of the digester to adopt suitable temperature and inoculum ratio for DAD of MSW.

## Results and discussion

### Effect of inoculum flow pattern on biogas production

**Figure 1:**
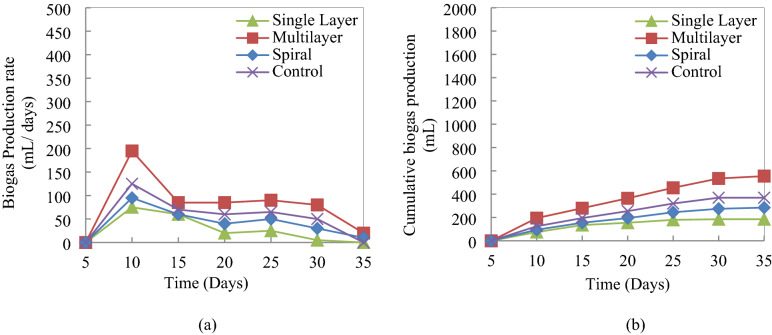
(**a**) Biogas production rate and (**b**) cumulative biogas production at single layer, multilayer, and spiral DAD reactor configurations and wet anaerobic digestion (WAD) reactor (control) at constant digestion temperature (25 °C) and CM inoculation.

The biogas production rates and cumulative biogas production from DAD of MSW for various inoculum flow patterns are illustrated in Fig. 1a,b, respectively. One of the major challenges with the DAD is the ineffective mass transfer between inoculum and feedstock material. Digesters with various configurations (described in “Inoculum flow pattern”) were designed so that the inoculum can reach the bulk feedstock at lowest possible (residence) time.

**Figure 2:**
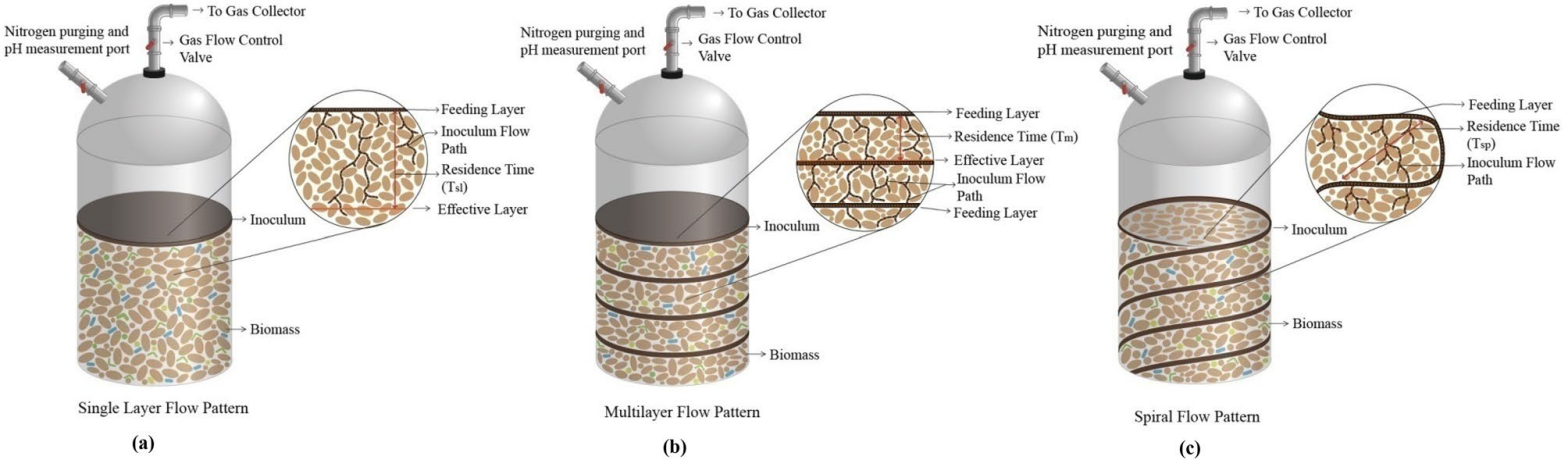
Residence time requirement for efficient mass transfer between different layers of biomass inside the various DAD reactor configurations. Effective layer is considered as a biomass layer where sufficient amount of biomass has been digested anaerobically to produce measureable amount of biogas.

Biogas production rate in all digesters followed a similar pattern. The biogas production increased initially, remained constant afterward, and finally reached the declining phase. The biogas production rate was highest in the multilayer flow pattern while it was lowest in single layer flow pattern. Residence time (T_sl_) was highest for the single-layer inoculum pattern (Fig. 2a) to reach from feeding layer to effective layer (a biomass layer where sufficient amount of biomass has been digested anaerobically to produce measureable amount of biogas) compared to residence time (T_m_) of the multilayer inoculum flow pattern (Fig. 2b). The highest residence time of the single-layer inoculum digester marked the slowest mass transfer between the inoculum and MSW biomass. This consequently resulted the lowest amount of biomass digestion followed by the lowest amount of biogas production in the single layer inoculum flow pattern^17,18^. On the contrary, due to inoculum feeding in several layers in a 4–5 cm interval throughout the biomass, mass transfer between the inoculum and biomass was improved significantly. As a result, the residence time (T_m_) requirement for the effective contact between them was the lowest in the multilayer flow pattern (Fig. 2b). Subsequently, the highest amount of biogas production was recorded in that digester due to the highest mass transfer rate. Because of the higher residence time (T_sp_) requirement for the spiral layer inoculum (Fig. 2c), an intermediate amount of biogas production rate was recorded among the different types of DAD process digester configurations. Submerged digester that functioned as control (also stand as WAD) has a relatively higher biogas production rate than the single and spiral layer inoculum flow pattern but a lower rate compared to the multilayer flow pattern. Due to submerged condition, the biomass to inoculum ratio became more inappropriate than the optimum value in the control digester, resulting a lower gas production rate than the multilayer inoculum flow pattern^19^. This lower gas production is also evident from the cumulative volume of biogas production recorded from the DAD and WAD (in control digester) in Fig. 1b. Multilayer inoculum flow pattern produced 555 mL biogas after 35 days of digestion while the WAD produced 370 mL biogas at the same time range. The gas production was lower in the other two types of inoculum flow patterns, single and spiral. For instance, spiral and single layer inoculum flow pattern produces 285 mL and 185 mL biogas respectively. Biogas production by varying digester configurations has been investigated by some other researchers also. Fagbohungbe, et al.^17^ reported 390 to 580 mL biogas production /kg VS of organic MSW while operating the digester in a continuous manner. In batch mode of digester operation, Fu et al.^18^ reported only (170–370) mL biogas production/kg VS. Lissens et al.^20^ optimized biogas production by operating digester in a semi-batch mode and reported different biogas yield than the previous authors. This variation could be explained by the different retention times used in the studies^17,21–23^.

### Effect of digestion temperature on biogas production

Optimization of inoculum flow pattern in the previous section was carried out at ambient or cryophilic temperature range. Since an increase in temperature enhances the biochemical activities of the microbes, digestion temperature was raised at the mesophilic temperature range^24^. The biogas production rate at every time point at the mesophilic range was higher than that for the digestion at ambient temperature (Supplementary Fig. S1a).This increasing trend is also further proved by the cumulative gas production recorded, as presented in Table 1. Cumulative gas production in the mesophilic temperature range was almost 3 times higher than the ambient temperature. The higher temperature increased the hydrolysis rate of the MSW. At the same time, acidogenesis was carried out at the proportional rate for the acetic acid generation which was then consumed at a similar rate through the methanogenesis step for the higher amount of biogas production. Several researchers^10,25–27^ have reported this trend. In a general observation, the biogas production is found almost doubled per 10 °C temperature increase in the mesophilic temperature range. In the current study also, the biogas production rate and cumulative biogas production was recorded more than double during anaerobic digestion at 37 °C compared to that at the ambient temperature of 25 °C (Table 1). Further increase in temperature to 40 °C did not increase the biogas production due to the unstable digestion process.. While running the digestion at 40 °C, higher energy input initially enhanced the MSW hydrolysis rate significantly that resulted accumulation of acetic acid within the digestion medium. Subsequently, it made the digestion medium acidic and negatively impact the methanogenesis^10,28^ Temperature increase in the mesophilic range from the ambient condition has no significant increase in the biogas production in case of submerged digestion. Biogas production is inhibited significantly in submerged conditions because of having a homogeneous digestion medium^29^. Homogeneous medium enhances the rapid mass transfer of acetic acid throughout the digestion medium resulted higher inhibition of biogas production in the submerged anaerobic digestion. The biogas production rate and cumulative biogas production decreased further with the increase in temperature to 37 and 40 °C. This phenomenon can also be attributed to the biochemical activities abatement in the fermentation broth^30–34^. However, cumulative gas production decreased by 54% and 40% for submerged fermentation and DAD respectively, when the temperature was increased from 37 to 40 °C (Table 1). It might be declined faster in submerged fermentation due to having a homogeneous fermentation medium that caused fast heat transfer rate. Contrary, because of the heterogeneous medium in DAD, the heat transfer rate was slower compared to submerged fermentation and caused a 40% decrease in cumulative gas production.

**Table 1:** Cumulative biogas yield at mesophilic temperature range.

Temperature (°C)	Cumulative gas production (mL)	
	Multilayer	Control
25	555	370
30	770	235
37	1515	175
40	900	80

### Effect of single and mixed mode of inoculation

**Figure 3:**
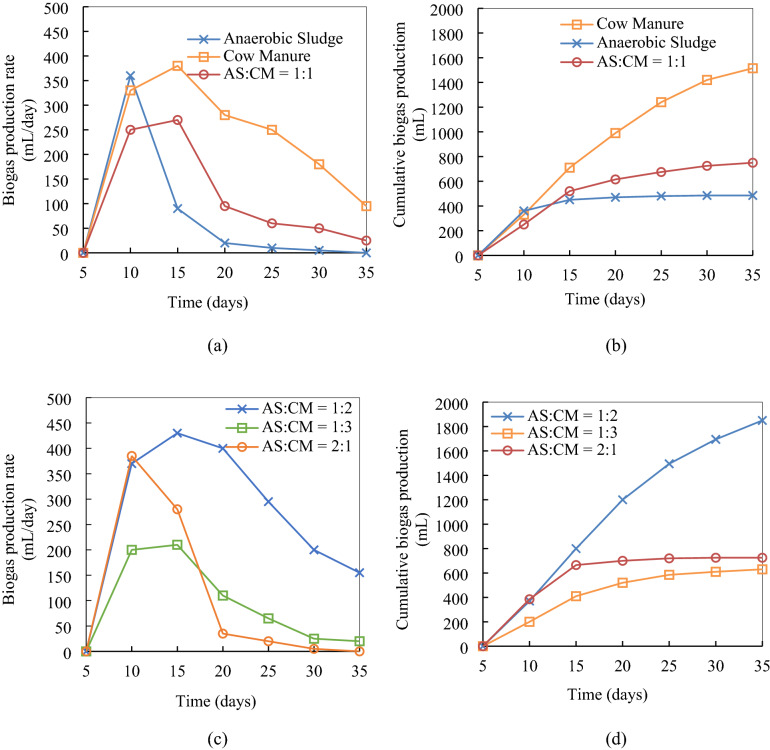
(**a**) Biogas production rate and (**b**) cumulative biogas production at single and mixed mode of inoculation (**c**) biogas production rate and (**d**) cumulative biogas production at varying mixed inoculum ratio. DAD conditions: multilayer inoculum flow pattern digester, 37 °C.

**Figure 4:**
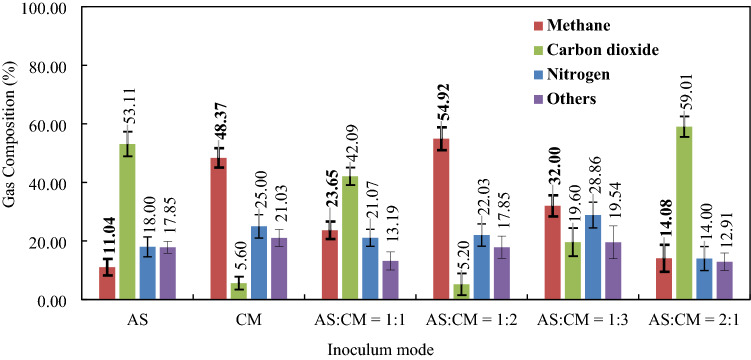
Biogas composition analysis at single and mixed mode of inoculation (gas produced other than the methane, carbon dioxide, and nitrogen are included in the “Others” in GC analysis). GC analysis conditions: column temperature—250 °C, pressure—375 kPa, and carrier gas (Helium) flow rate—20 mL/min.

Single and mixed-mode inoculation was used in this study to observe their effects on biogas production. A 5-day period was allowed for the inoculum to adjust with the digestion environment. The cow manure (CM) inoculation showed the higher amount of biogas production rate between 5 to 15 days (Fig. 3a). Afterward, it started to decrease gradually up to 95 mL/day, however, the rate was higher than all other inoculation modes throughout the fermentation period (35 days). Several researchers^35–38^ have reported that CM enriched with light metals (Na^+^, K^+^, Mg^2+^, Ca^2+^)^39^. These trace metals maintain the metabolic osmotic pressure and work as enzyme cofactor constituents for the digestion medium. The absence of light metals reduced the anaerobic biogas production in several previous studies^40,41^ while some other authors reported a breakdown of the whole digestion process in their absence^42^. So, the trace metals are commonly added externally in the digestion medium containing low nutrient levels^42,43^. Moreover, periodic pH measurement during the digestion cycle showed pH 6.8–8.0 that is suitable for both acetogenic and methanogenic bacteria^44^. A higher activity of methanogenic bacteria was also confirmed by the cumulative biogas production (Fig. 3b) and its composition analysis (Fig. 4). Due to this better performance, 48.37% (v/v) methane content was achieved in the 1515 mL cumulative biogas (Fig. 3b) by CM inoculation. With the same inoculum usage, only 5.60% (v/v) CO_2_ was produced since most of the produced CO_2_ in the hydrolysis and acidogeneis steps were converted into methane.

On the other hand, the pH values were observed 5.8 to 7.0 range when anaerobic sludge (AS) was used for MSW digestion. In this pH range, both hydrolytic and acidogenic bacteria work better with the fast doubling time and growth rate^45,46^. Therefore, the highest biogas production of 360 mL/day was recorded between 5 and 10 days period in this mode of inoculation (Fig. 3a) which was even higher than initial biogas production from the CM inoculum—330 mL/day. But this higher biogas production did not sustain beyond 10 days of digestion since the pH (5.8–7.0) in the AS mode of inoculation was not suitable for the subsequent steps of the anaerobic digestion, acetogenesis, and methanogenesis. Unfavorable pH also resulted a lower percentage of methane production, 11.04% (v/v) (Fig. 4). However, a higher percentage of CO_2_ production (53.11% (v/v)) confirms the initial higher activity of hydrolytic and acidogenic bacteria. Furthermore, AS inoculum used in this study contains a lower fraction of light metals but contains heavy metals such as Cu^2+^, Zn^2+^, Cd^2+^, Pb^2+^ etc. at higher concentrations^47–53^. Those heavy metal ions have both inhibition and toxic effects on acetogenic and methanogenic bacteria, resulting interruption in enzyme secretion (lower activities of both types of bacteria)^39,54–56^. Ultimately, the digestion process with the AS inoculum collapsed (no biogas production) at 25 days and onwards (Fig. 3a,b) due to VFAs accumulation. As discussed earlier the lack of light metals in the digestion medium but the presence of heavy metals at the inhibitory concentrations could collapse the total digestion process^57^. This fact is observed in the present study where heavy metals (Cu^2+^, Zn^2+^, Cd^2+^, Pb^2+^ etc.) in the AS inoculum inhibitthe biogas production from the beginning which has been stopped totally after 25 days. In summary, due to arid conditions and unfavorable pH range for acetogenic and methanogenic bacteria, a higher initial biogas production rate decreases sharply below 100 mL/day and reaches to 0 mL/day before the total digestion period. However, while CM is mixed with the AS at 1:1 ratio, its nutrient conditions and pH values reach in suitable conditions for the anaerobic digestion. Therefore, biogas production rate and methane content at this inoculum mixer (AS:CM = 1:1) were improved than the single AS inoculum. Additionally, cumulative biogas production (750 mL) was observed lower than using CM (1515 mL) only and higher than using AS only (485 mL) as inoculum (Fig. 3b). Similarly, methane content (23.65%) was also remained intermediate position compared to the single mode of inoculation—AS (11.04%) and CM (48.37%) (Fig. 4).

### Biogas production from the variation of inoculum proportion in mixed inoculum

It was observed (from the previous “Effect of single and mixed mode of inoculation”) that CM addition with the AS in the mixed inoculum enhances biogas production due to nutrient enrichment. Three inoculum blends (AS:CM = 1:2, 1:3, and 2:1) were investigated to determine their effect on the system performance. Among all the mixtures, AS:CM ratio 1:2 resulted in the highest biogas production rate (Fig. 3c). This could be attributed to a high metal nutrient addition from the CM inoculum^39,58–60^. Additionally, an optimal pH (6.8–8.0) for the anaerobic digestion could also be responsible for this higher amount of biogas production. But initially measured pH was comparatively lower (6.0–6.5) which indicated higher hydrolysis and acidogenesis rates due to the VFAs generation at earlier stages. Higher VFAs generation between 10 and 20 days of digestion period due to lower pH was also justified by the Supplementary Fig. S2. Further periodic records of pH showed higher values of 6.8–8.0 after 20 days. This higher pH has probably resulted from the rapid consumption of VFAs which were generated in previous stages. Therefore, VFAs generated with the inoculum AS:CM = 1:2 might be anticipated to be acetic acid which was readily converted through the methanogenesis step and generates alkalinity (pH 8.0)^61^. Alkalinity above pH 8.0 could have resulted from the ionic form of hydrogen sulfide and ammonia^62^. Higher alkalinity disintegrates the microbial communities and ultimately stops the digestion process. However, pH was measured at the optimal range (6.8–8.0) between 20 and 35 days of the final digestion stages in the present study. This suitable pH range (6.8–8.0) enhanced the activities of both methanogenic bacteria—acetoclastic methanogens (utilize acetic acid for methane production) and hydrogenotrophic methanogens (utilize hydrogen and CO_2_ for methane production)^44,63,64^. Consequently, the highest methane—54.92% (v/v)—and lowest CO_2_ yield—5.20% (v/v)—were observed with this mixed inoculum among all the single and mixed inoculation (Fig. 4).

Interestingly, further increase in the CM fraction (AS:CM ratio 1:3) resulted in a decrease in both cumulative biogas production (630 mL in Fig. 3d) as well as the methane content (32% (v/v) in Fig. 4). This behavior seems strongly connected to theperiodic pH changes. Initial p H(5.8–6.0) with this inoculum mixture was lower than the previous inoculum mixture (AS:CM = 1:2). This lower pH ascribed faster hydrolysis and acidogenegis rate. On the high pH values of 6.5–7.3, the later stages of the process (20–30 days) were complemented with the lower methane production (32% (v/v)) (Fig. 4) and slow VFAs conversion rate (Supplementary Fig. S2). It could be attributed to the presence of propionic and butyric acids at higher concentrations in the generated VFAs, which have been reported to yield lower conversions compared to the acetic acid^65,66^. Moreover, lower methane production could also be a result of the high C/N ratio attained due to high amount of CM addition. The C/N ratio of 20–30 is considered optimum for the AD process^67^. However, as the CM portion was increased (at AS:CM = 1:3), the C/N ratio increased beyond the optimum value, resulting in the lack of nitrogen for their growth^68^. Lower inoculum cell growth resulted in less amount of biogas production in the AS:CM = 1:3 inoculum ratio. While AS ratio was increased, AS:CM = 2:1, in the mixed inoculum, lower pH value 5.0–5.5 was recorded in the digestion medium which was most suitable for the first two stages—hydrolysis and acidogenesis—of anaerobic digestion^69^. Due to higher rates of hydrolysis and acidogenesis, VFAs and CO_2_ (59.01% (v/v)) were observed higher (Supplementary Fig. S2 and Fig. 4). But the next stages of anaerobic digestion such as acetogenesis and methanogenesis were not carried out at a significant rate which pointed out the accumulation of large amount of VFAs (5700 mg/L) in the digestion medium. Subsequently, an imbalanced situation between generation and intake of VFAs was happened resulted in irreversible acidification in the digestion medium. This irreversible acidification inhibited biogas production rate (Fig. 3c), cumulative biogas production (Fig. 3d), and ultimately methane yield (14.08% (v/v)) (Fig. 4) with this mixed inoculum ratio (AS:CM = 2:1). On the contrary, a proportionate amount of VFAs generation and subsequent consumption was recorded in AS:CM = 1:2 inoculum mixer (Supplementary Fig. S2), resulting the highest amount of biogas production (1850 mL in Fig. 3d) with the highest methane production 1256.58 mL CH_4_/kg VS (Supplementary Table S2).

The biogas (2288.02 mL/ kg VS) and methane (1256.58 mL/kg VS) yield observed in this study are within the range of values reported in other DAD studies (Supplementary Table S3). The values are relatively lower compared to the previous study using MSW as a feedstock (120–200 L/kg VS). This could be the result of various factors but the main factor is the type of MSW. The previous study used the sorted organic fraction of the biomass; no plastic, metal, and other non-biodegradable materials were present in the digestion process. Although this sorting improves the quality of feedstock, however, the associated costs limits the commercial implementation of the process. the Secondly, to improve the biogas yield, authors utilized leachate/digestate recirculation around the digester^70^ while some others reported the pre-aeration of the MSW feedstock. These additional recirculation or pre-aeration again incurs the additional operating and capital cost for the biogas production. In contrast, the current study kept the digester undisturbed for the entire digestion period while tried to improve the biogas yield by varying the digester configurations. In addition, higher F/I ratio (about 20) were used those processes for increased biogas yield which increased the risk of biohazards associated with the higher amount of inoculum usage as well as higher cost for this large amount of inoculum. On the contrary, current study reduced such hazards and costs by using F/I ratio at 2. Moreover, Supplementary Table S3 shows that DAD processes maintained digestion period between 100 and 200 days for higher biogas yields. Long digestion period would result higher operating cost for the biogas production and preservation problem of MSW, but the present study only used the 35 days of digestion period.

While lignocellulosic waste materials were used in the DAD process as feedstock (Supplementary Table S3), methane yields were reported as 305–335 mL/kg VS^71,72^. Those yields are mostly lower than the calculated yields of the present study. Moreover, lignocellulosic feedstock-based DAD processes used various biomass pretreatment techniques, such as alkali treatment, acid treatment, and hydrothermal treatment, which are energy-intensive and expensive^73,74^. Without using any pretreatment processes, the present study generated higher biogas yields compared to the lignocellulosic waste-based DAD processes. One poultry manure-based DAD processes reported relatively higher methane yields (50–180 L/kg VS) than the current study^75^. Those processes used another manure, for example, pig manure, as a inoculum which increased the risk of imbalanced C/N ratio throughout the digestion period. Therefore, manure-based DAD processes used the continuous stirred tank reactor (CSTR) which has higher operating and capital costs and operational complexity than the simple batch digester used in the present study^76^.

### Limitations of this DAD study

Although the overall results are highly promising, there are some limitations associated with the DAD process yet to be addressed for its large-scale application. Most of the studies, including the current study, used lab-scale digesters with a shorter digestion period. The biogas generation at augmented digestion period beyond the 35 days, scaled up digester volume, etc. has not been tested yet. Comprehensive techno-economic analysis studies are also needed to understand the commercial-scale viability and competitiveness of the process. A number of techno-economic assessments of submerged anaerobic digestion have been reported^77^, but there is a lack of study on DAD for biogas production, particularly in large scale. Similarly, a life cycle assessment (LCA) study is critical to understand the environmental benefits of the process compared to the conventional AD processes and the current MSW management practices. Except for common features of LCA for biogas technology, focal points of DAD may include (i) impact on the natural environment of municipal or agriculture wastes collection and sorting, (ii) freshwater use, energy input, emissions to air, wastewater production, (iii) land use for upstream and downstream processing, (iv) injurious level to human health, and (v) environmental impacts due to co-products management. The integrated TEA-LCA studies on DAD could contribute to its further sustainable improvement. Moreover, produced biogas from this study is not suitable for commercial applications since it contains 54.92% (v/v) methane. Methane content of the biogas should be more than 80.00% to be used as a biofuel, for example, as a gaseous transportation fuel and addition to national gas grid lines^78,79^. However, Biogas with this methane percentage (54.92% (v/v)) is still useful for cooking and heating purpose. Another limitation of the study is that no remediation technologies have been investigated to tackle the digestate of the DAD process. The follow-up studies focus on this aspect through hydrothermal carbonization (HTC) of the digestate to produce hydrochar for either solid fuel or biofertilizer production (based on the quality analysis of the biochar).

## Materials and methods

### Digester fabrication

**Figure 5:**
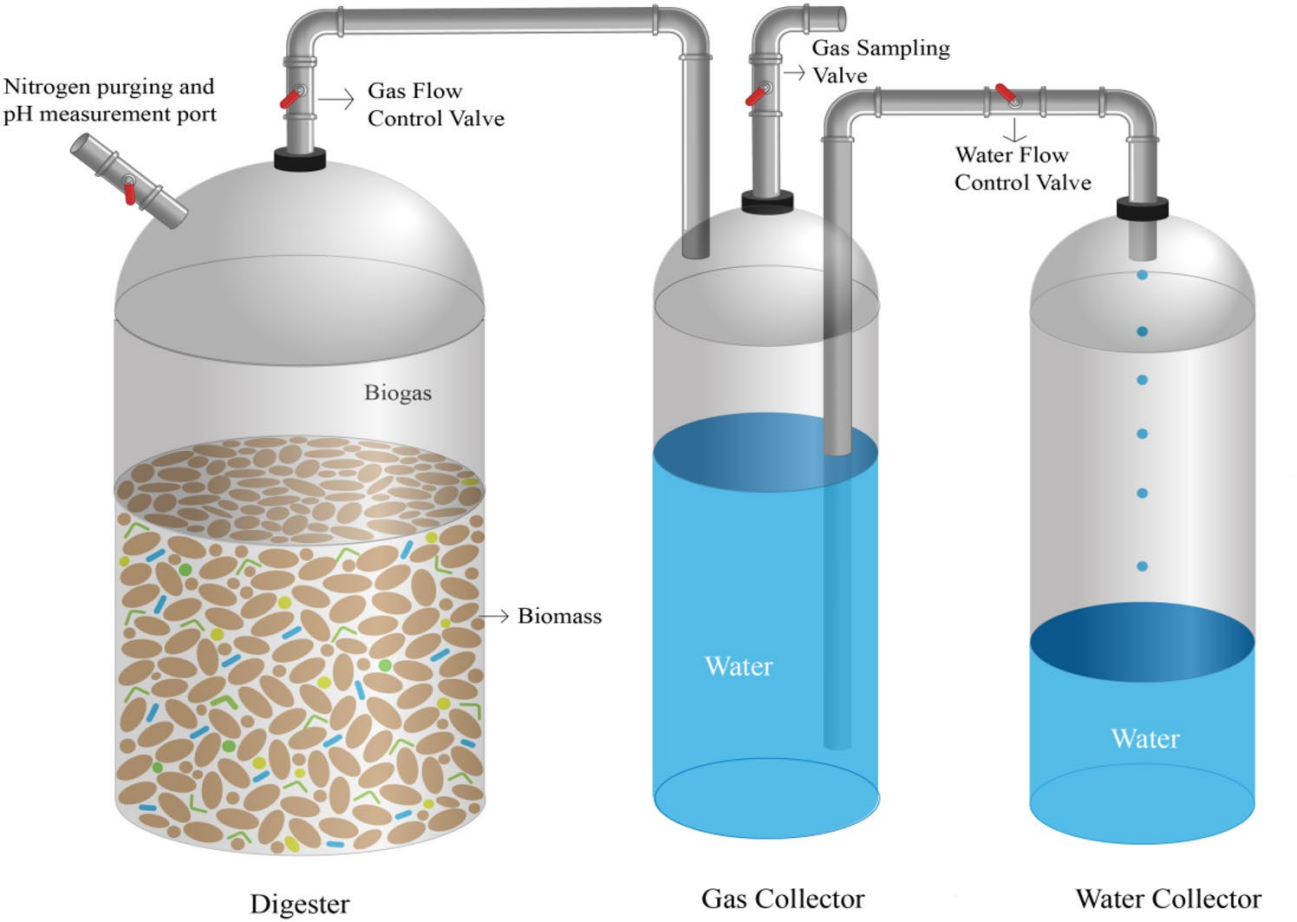
Simple schematic of set-up used for the DAD process. AS and CM were used as inoculum at the single and mixed mode of inoculation in the “Digester” at the single layer, multilayer, and spiral inoculum flow pattern. “Gas Collector” and “Water Collector” were used in the water displacement method for produced biogas and displaced water collection correspondingly.

Several laboratory-scale digesters were fabricated in this study to carry out the DAD process. Polyethylene terepthalate (PET) plastic material was used for each reservoir and digester construction. The total volume of the digester (Fig. 5) was 3.0 L while the working volume was 2.5 L. Digester was connected to a gas collector (a 2.0 L PET reservoir initially filled with water) by a flexible plastic pipe. DAD process produced biogas in the digester and the volume of the generated biogas was measured by the water displacement method. Biogas volume was recorded in a 5 days interval to measure the biogas production rate. Nitrogen gas was purged inside the digester after biomass loading to make the digestion environment oxygen free. The pH of the digestion environment was also monitored periodically using the nitrogen purging port. It was ensured that the complete system was air tight. An external water bath was used to maintain the constant digestion temperature.

### Waste collection, processing, and MSW feedstock preparation

A model MSW sample was prepared by mixing the domestic kitchen waste (75%), plastic (8.5%), paper (7%), fabrics (3.5%), and other waste (glass and metals) (6%) in a ratio that is commonly observed in the cited literature (Supplementary Table S4)^80,81^. The kitchen waste, mostly containing vegetable waste, waste rice, waste meat, bones, fish waste, eggshell, was collected from the student’s dormitory and nearby teacher and officer’s dormitory at the Shahjalal University of Science and Technology (SUST), Sylhet, Bangladesh. A 900 g of organic kitchen waste was used in each DAD experiment. Post-consumer PET, Polyethylene, Polyvinyl Chloride (PVC), and Polypropylene (PP) plastics, and paper were collected from the same university’s waste collection bins. A total of 100 g plastics and 85 g paper were used in each experiment. Similarly, fabric (40 g), glass (40 g), and metal (35 g) wastes were collected, sized into 1–2 cm, and mixed according to the proportions mentioned above; making the total substrate amount 1200 g (dry weight basis). Volatile solid (VS) content of the model feedstock was measured 67.38% (dry basis), following Laboratory Analytical Procedure for determination of VS content in biomass developed by NREL.

### Inoculum preparation

AS and CM were used as inoculum in a single and mixed-mode in this study. VS content was measured 47.50% (dry basis) and 73.20% (dry basis) for AS and CM accordingly. AS was collected from the sewerage drain of the SUST and CM was collected from a cattle farm close to the university. Then collected AS and CM were used in different amounts and ratios (specified in Tables 2 and 3) in 500 mL water to form inoculum slurry. The inoculum was prepared in slurry condition so that it can flow easily through the biomass. Feedstock to inoculum (F/I) ratio was always maintained at 2 (based on VS content) in this study because several other authors reported this as optimum F/I ratio for DAD^82,83^.

**Table 2:** Experimental design for DAD process inoculum flow pattern optimization using AS and CMin single or mixed of inoculation and temperature optimization in the mesophilic and thermophilic range.

Digestion process parameter	Inoculum flow pattern optimization				Temperature optimization			
Inoculum flow pattern	Multilayer	Spiral	Single-layer	Control digester	Opt. FP^a^ (multilayer)	Opt. FP^a^ (multilayer)	Opt. FP^a^ (multilayer)	Submerged condition
Temperature (°C)	25	25	25	25	30 °C	37 °C	40 °C	Control digester at varying temperature
Amount of Biomass (g)	1200	1200	1200	1200	1200	1200	1200	1200
Amount of NaOH (mL)	50	50	50	0	50	50	50	0
Amount of added water (mL)	0	0	0	1000	0	0	0	1000
Amount of inoculum^b^ (g)	552.30	552.30	552.30	552.30	552.30	552.30	552.30	552.30
Nitrogen purging (min)	5	5	5	5	5	5	5	5

^a^Optimized flow pattern after the inoculum flow pattern optimization was used for temperature optimization.

^b^Inoculum was added in 500 mL water during DAD process.

**Table 3:** Experimental design for inoculum type and ratio optimization (Digester configuration: multilayer inoculum flow pattern, and digestion temperature: 37 °C).

Digestion process parameter	Single and mixed mode of inoculation					
	CM	AS	AS:CM (1:1)	AS:CM (1:2)	AS:CM (1:3)	AS:CM (2:1)
Amount of Biomass (g)	1200	1200	1200	1200	1200	1200
Amount of NaOH (mL)	50	50	50	50	50	50
Inoculum Flow Pattern^a^	Opt. FP (multilayer)	Opt. FP (multilayer)	Opt. FP (multilayer)	Opt. FP (multilayer)	Opt. FP (multilayer)	Opt. FP (multilayer)
Amount of inoculum^b^ (g)	552.30	552.30	276.15 : 425.56	283.71: 368.20	212.78:414.22	567.41:184.10
Digestion temperature^c^ (°C)	Opt. T (37)	Opt. T (37)	Opt. T (37)	Opt. T (37)	Opt. T (37)	Opt. T (37)
Nitrogen purging (min)	5	5	5	5	5	5

^a^Optimized flow pattern from the “Inoculum flow pattern”.

^b^Inoculum was added in 500 mL water during DAD process.

^c^Optimized temperature from the “DAD temperature”.

### Experimental run

In a typical experimental run, 50 mL NaOH solution (1.5% w/w) was mixed thoroughly with the MSW biomass feedstock and then loaded inside the digester. This alkaline solution was added to lower the retention time and maintain the optimum pH (7.0–8.0) of the digestion process^84^. The inoculum was introduced at the various manner in single and mixed mode throughout the biomass, as described in the next section. The complete system setup was sealed and nitrogen was purged (for 5 min at 1.5 L/min) into the digester to create an anaerobic atmosphere for the digestion. Lastly, the sealed anaerobic digester was placed in a water bath so that a fixed digestion temperature can be maintained throughout the 35 days of digestion period. In this total period, the volume of produced biogas was measured by the water displacement method (described in “Digester fabrication”) and recorded in a 5 days interval. Finally, biogas produced in the whole digestion period was collected in the Tedlar^®^ gas sampling bag. Subsequently collected biogas composition analysis was carried out by Gas Chromatograph (GC) (Model 2014B, Shimadzu, Japan) equipped with a flame ionization detector (FID), solid phase: polyethylene glycol (fused silica capillary column − 15 m × 0.25 mm × 0.25 µm film thickness) and carrier gas was helium. In GC analysis, following conditions were used for the methane, nitrogen, and carbon dioxide content measurement: column temperature—250 °C, pressure—375 kPa, and constant carrier gas flow rate—20 mL/min). Additionally, volatile fatty acids (VFAs) concentration was measured by two-step titration method during the mixed mode of inoculation^85,86^.

### DAD process parameter optimization

#### Inoculum flow pattern

Since DAD requires a long retention time compared to submerged digestion, various digester configurations were investigated to reduce the retention time (schematics are shown in Supplementary Fig. S3). Digesters with different possible types of inoculum flow patterns, single layer, multi-layer, and spiral, were constructed for effective contact between inoculum and biomass at ambient temperature (25 °C). In the first configuration, all the CM inoculum was placed at the top of the biomass as a single layer, referred as single layer inoculum flow pattern. In the second configuration, the inoculum was placed in several layers in a 4–5 cm interval throughout the biomass, referred as multilayer inoculum flow pattern. In the third configuration, the inoculum was flowed by a spiral and flexible plastic pipe throughout the biomass and this inoculum flow pattern was referred as spiral flow pattern in this paper. A control digester was also run at the submerged anaerobic condition to compare the performances of the two approaches. Overall, the experimental design to find out the optimum inoculum flow pattern for DAD is summarized in Table 2.

#### DAD temperature

The optimized inoculum flow pattern from “Inoculum flow pattern” was used to observe the effect of digestion temperature on biogas production. In addition to ambient temperature (used in “Inoculum flow pattern”), three other temperatures (30 °C, 37 °C, and 40 °C) were selected to determine the optimum value using only CM as inoculum. The experimental conditions for this specific task are also presented in Table 2. A control fermenter at each temperature was also run to compare the biogas yield from dry and submerged anaerobic digestion of MSW.

#### Inoculum type

Three types of inoculum (AS, CM and a mixer of AS and CM) were investigated to determine the inoculum producing the highest amount of biogas in DAD process. The experimental design for this inoculum optimization is summarized in Table 3. Optimized conditions from “Inoculum flow pattern” and “DAD temperature” were used in this experimental design.

#### Mixed inoculum ratio

The mixed inoculum was flowed at various ratios (based on their VS content) to find the variation of amount of each inoculum on the biogas production. AS and CM were mixed at 1:2, 1:3, and 2:1 ratios and the experimental design is presented in Table 3.

## Conclusion

Summary of the study could be concluded as

(i) Multilayer inoculum flow pattern can effectively increase the biogas yield compared to the single and spiral layer inoculum flow pattern. The lowest biogas yield was recorded 185 mL with the single-layer inoculum configuration, whereas 285 mL biogas yield was achieved in the spiral layer digester. In contrast, 1.95 and 3.0 times higher biogas was produced in the multilayer inoculum flow pattern than the spiral and single layer inoculum flow pattern respectively.
(ii) Constant mesophilic digestion temperature (37 °C)—is suitable for biogas production than the ambient and thermophilic digestion temperature. A 37 °C digestion temperature increased the biogas production to 1515 mL from 770 mL (at 30 °C) and 900 mL (at 40 °C) due to enhanced microbial activities for proportional amount of VFAs generation and consumption.
(iii) Mixed inoculum can enhance both biogas yield and methane content compared to single inoculum if a proper digestion environment such as pH, nutrient level etc. is maintained. pH in 6.8–8.0 range lowers the VFAs accumulation and increases the hydrolysis of the MSW feedstock, resulting higher biogas yield. Similarly, presence of light metals (Na^+^, K^+^, Mg^2+^, Ca^2+^) enhance the biochemical activities for accelerated biogas generation.
(iv) Mixed inoculum at the AS:CM = 1:2 ratio produces the highest amount of cumulative biogas with the highest methane content, 54.92% (v/v), and methane yield, 1256.58 mL CH_4_/kg VS.

## Supplementary Information

Supplementary Information.
