# Supplementary material for: Impact of temperature, inoculum flow pattern, inoculum type, and their ratio on dry anaerobic digestion for biogas production
Source: Sci Rep. 2022 Apr 13;12:6162. doi: 10.1038/s41598-022-10025-1 (PMC9007994; doi:10.1038/s41598-022-10025-1)
Supplement: Supplementary file 1 — Supplementary Information. [file 41598_2022_10025_MOESM1_ESM.pdf]

Impact of temperature, inoculum flow pattern, inoculum type, and their ratio on dry anaerobic digestion for biogas production

Md Shahadat Hossain[a,b], Tahmid ul Karim[b], Mahade Hassan Onik[b], Deepak Kumar[a], Md Anisur Rahman[b,c], Abu Yousuf[b,*], Mohammad Rakib Uddin[b,d]

[a]Department of Chemical Engineering, State University of New York College of Environmental Science and Forestry, Syracuse, NY 13210, USA

[b]Department of Chemical Engineering and Polymer Science, Shahjalal University of Science and Technology, Sylhet - 3114, Bangladesh

[c]Department of Chemical Engineering, University of Massachusetts Amherst, Amherst, Massachusetts, USA

[d]Dipartimento di Ingegneria, Università degli studi di Napoli "Parthenope", Napoli – 80143, Italy

Supplementary Table S1: Commercially available DAD digester (**SS-OFMSW**: source sorted organic fraction of municipal solid waste, **OFMSW**: Organic fraction of municipal solid waste, **OW**: Organic waste, **NA**: Not available).

**Digestion parameter**	**Digester type**				
	DRANCO [5]	KOMPOGAS [5]	VALORGA [6]	BEKON [7]	BIOFerm [7]
Operation mode	Continuous	Continuous	Continuous	Batch	Batch
Digestion temperature (^{o}C)	50-55	55	37-55	40-55	37
Biomass feedstock	SS –OFMSW	OFMSW	OFMSW	OW	OFMSW
TS (%)	20-40	30	30-60	40	25
SRT (days)	20	29	20-33	28-35	28
OLR (kg VS/m^3/d)	10 -15	4.3	10-15	NA[e]	NA
VS removal	40-70	60-70	60-65	65-70	50-55
Methane yield (m^3/kg VS)	0.21-0.30	0.39-0.58	0.21-0.30	0.17-0.37	0.21-0.35
Origin	Belgium	Switzerland	France	Germany	Germany

Supplementary Table S2: Cumulative biogas and methane production at various AS and CM mixed inoculum ratio during multilayer inoculum flow pattern digestion at 37 °C.

Mixed Inoculum ratio (AS: CM)	Cumulative Gas Production (mL)	Methane production ($mL\ CH_4/kg\ VS$)
1:1	750	219.37
1:2	1850	1256.58
1:3	630	249.33
2:1	725	125.53

Supplementary Table S3: Comparative analysis of the previous DAD studies with the present study for methane and biogas yield analysis

Feedstock		Dry fermentation conditions	Methane yield (mL / kg VS)	Biogas yield (mL / kg VS)	Reference
MSW	Unsorted biomass	37 °C, pH (6.8 - 8.0), cow manure and anaerobic sludge (single and mixed mode inoculum), multilayer batch digester, 35 days	219.37<br>1256.58<br>249.33<br>125.53	927.57 (1:1) *<br>2288.02 (1:2)<br>779.16 (1:3)<br>896.66 (1:4)	This study
MSW	Organic fraction	35 °C, OLR 4.55 kgVS/m^3 day, liquid digestate recirculation	140×10^3	-	Di Maria *et al.*[8]
	Organic fraction	Pre-aeration, pH 7.8, anaerobic sludge (inoculum), leachate recirculation, ~100 days	-	192.4×10^3	Ni *et al.*[9]
	Mechanically sorted organic fraction	40 °C, pH 6.2, 200 days, F/I 17	139×10^3	-	Basinas *et al.*[10]
	Organic fraction	55 °C, OLR (7–10) kgVS/m^3, 280 days, cow manure and anaerobic sludge mixture (inoculum)	$(121 – 327) \times 10^3$	-	Rocamora *et al.*[6]
Lignocellulosic biomass	Corn stover	Alkali pretreatment, 38 °C, pH 7.5, batch digester, pig manure (inoculum)	335.00	-	Liu *et al.*[11]
	Sugarcane bagasse	Hydrothermal and alkali pretreatment, 37 °C, batch digester, 35 days, anaerobic sludge (inoculum), F/I 0.5	318.00	-	Mustafa *et al.*[12]
	Sorghum bagasse	Alkali and H_2O_2 pretreatment, 37 °C, pH 8.16, cow and chicken manure mixture (inoculum), batch digester, F/I 1.5	330.00	-	Cao *et al.*[13]
	Wheat straw	Urea pretreatment, 35 °C, 120 rpm, activated sludge (inoculum)	305.50	-	Yao *et al.*[14]
Poultry manure	Chicken manure	37 °C, continuous stirring tank reactor (CSTR), pig manure (inoculum), ~100 days	180.00×10^3	-	Bi *et al.*[15]
	Swine manure	Thermal pretreatment, 35 °C, CSTR, pig manure (inoculum) , 41 days	50.00×10^3	-	Hu *et al.*[16]

*mixed inoculum ratio

Supplementary Table S4: Composition of MSW generated across the major cities of Bangladesh and prepared feedstock for the DAD study.

Waste fraction	Waste percentage [17,18] (%)	Waste percentage [19,20] (%)	Prepared MSW for this study (%)
**Kitchen Waste**	75.64	63.74	75
**Plastic**	8.45	8.07	8.5
**Paper**	7.22	6.24	7
**Fabrics**	3.45	2.05	3.5
**Others**	5.24	19.9	6
**(Glass, Metals, etc.)**			
**Volatile solid (%) (dry basis)**			67.38
**Size (cm)**			1 – 2

Supplementary Information 1

CH$_4$ production calculation equation:

$$CH_4 \text{ production } \left(\frac{\text{mL } CH_4}{\text{kg VS}}\right) = \frac{\text{Cumulative biogas production (mL)} * \text{Methane content\% (v/v)}}{\text{Amount of biomass (gm)(dry basis)} * \text{VS content\% (dry basis)}} * \frac{1000 \text{ gm}}{1 \text{ kg}}$$

CH$_4$ production calculation at mixed inoculum ratio:

at AS:CM = 1:1 ratio:

$$CH_4 \text{ production } \left(\frac{\text{mL } CH_4}{\text{kg VS}}\right) = \frac{750 \text{ mL } * 23.65\%}{1200 \text{ gm } * 67.38\%} * \frac{1000 \text{ gm}}{1 \text{ kg}} = 219.37 \ \frac{\text{mL } CH_4}{\text{kg VS}}$$

at AS:CM = 1:2 ratio:

$$CH_4 \text{ production } \left(\frac{\text{mL } CH_4}{\text{kg VS}}\right) = \frac{1850 \text{ mL } * 54.92\%}{1200 \text{ gm } * 67.38\%} * \frac{1000 \text{ gm}}{1 \text{ kg}} = 1256.58 \ \frac{\text{mL } CH_4}{\text{kg VS}}$$

at AS:CM = 1:3 ratio:

$$CH_4 \text{ production } \left(\frac{\text{mL } CH_4}{\text{kg VS}}\right) = \frac{630 \text{ mL } * 32.00\%}{1200 \text{ gm } * 67.38\%} * \frac{1000 \text{ gm}}{1 \text{ kg}} = 249.33 \ \frac{\text{mL } CH_4}{\text{kg VS}}$$

at AS:CM = 2:1 ratio:

$$CH_4 \text{ production } \left(\frac{\text{mL } CH_4}{\text{kg VS}}\right) = \frac{725 \text{ mL } * 14.00\%}{1200 \text{ gm } * 67.38\%} * \frac{1000 \text{ gm}}{1 \text{ kg}} = 125.53 \ \frac{\text{mL } CH_4}{\text{kg VS}}$$

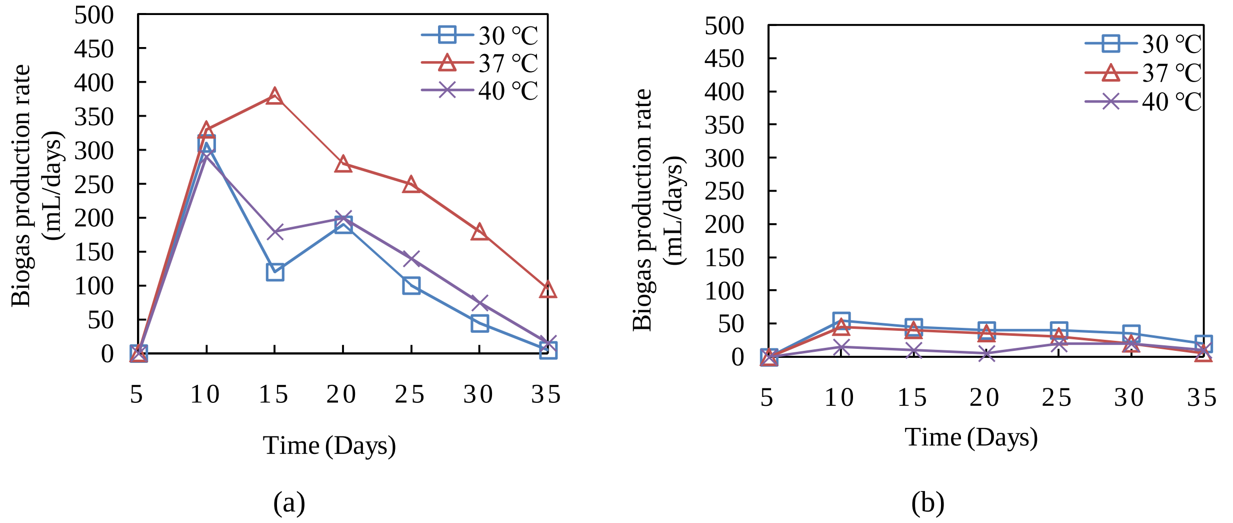

Supplementary Figure S1: Biogas production rate at mesophilic temperature range for (a) multilayer inoculum flow pattern and (b) control digester (at submerged condition)

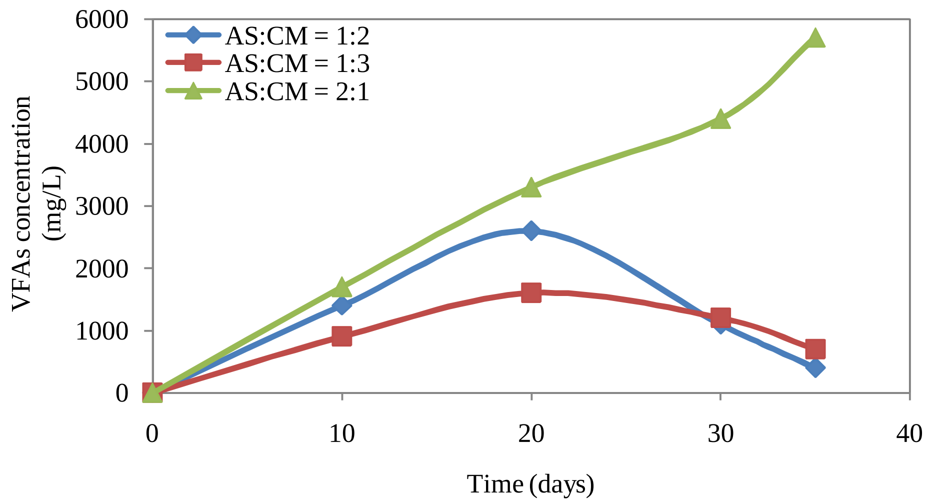

Supplementary Figure S2: VFAs generation and consumption in mixed mode of inoculation during DAD (Digestion conditions: multilayer inoculum flow pattern, 37 ℃, AS and CM mixed inoculum

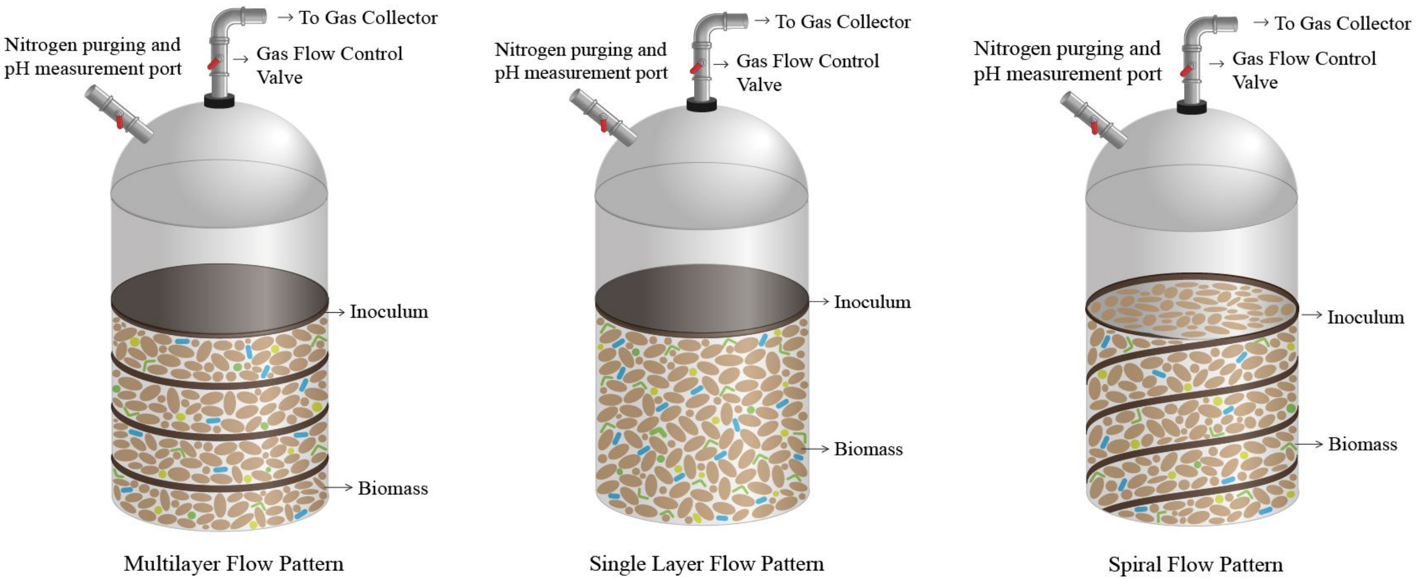

Supplementary Figure S3: Digester configurations with varied inoculum flow patterns (single layer flow pattern - all the CM inoculum was placed at the top of the MSW, multilayer flow pattern - inoculum was placed in several layers in a 4-5 cm interval throughout the MSW, and spiral flow pattern - inoculum was flowed by a spiral and flexible plastic pipe throughout the MSW biomass).

References

1 Kiyasudeen, K., Ibrahim, M. H., Quaik, S. & Ismail, S. A. in *Prospects of organic waste management and the significance of earthworms* 23-44 (Springer, 2016).

2 Kothari, R., Pandey, A., Kumar, S., Tyagi, V. & Tyagi, S. Different aspects of dry anaerobic digestion for bio-energy: An overview. *Renewable and Sustainable Energy Reviews* **39**, 174-195 (2014).

3 Parkin, G. F. & Owen, W. F. Fundamentals of anaerobic digestion of wastewater sludges. *Journal of environmental engineering* **112**, 867-920 (1986).

4 Karthikeyan, O. P. & Visvanathan, C. Bio-energy recovery from high-solid organic substrates by dry anaerobic bio-conversion processes: a review. *Reviews in Environmental Science and Bio/Technology* **12**, 257-284 (2013).

5 Fagbohungbe, M. O. *et al.* High solid anaerobic digestion: Operational challenges and possibilities. *Environmental Technology & Innovation* **4**, 268-284, doi:https://doi.org/10.1016/j.eti.2015.09.003 (2015).

6 Rocamora, I. *et al.* Dry anaerobic digestion of organic waste: A review of operational parameters and their impact on process performance. *Bioresource Technology* **299**, 122681, doi:https://doi.org/10.1016/j.biortech.2019.122681 (2020).

7 Fu, Y. *et al.* Dry Anaerobic Digestion Technologies for Agricultural Straw and Acceptability in China. *Sustainability* **10**, doi:10.3390/su10124588 (2018).

8 Di Maria, F., Barratta, M., Bianconi, F., Placidi, P. & Passeri, D. Solid anaerobic digestion batch with liquid digestate recirculation and wet anaerobic digestion of organic waste: Comparison of system performances and identification of microbial guilds. *Waste Management* **59**, 172-180, doi:https://doi.org/10.1016/j.wasman.2016.10.039 (2017).

9 Ni, Z., Liu, J. & Zhang, M. Short-term pre-aeration applied to the dry anaerobic digestion of MSW, with a focus on the spectroscopic characteristics of dissolved organic matter. *Chemical Engineering Journal* **313**, 1222-1232 (2017).

10 Basinas, P., Rusín, J. & Chamrádová, K. Dry anaerobic digestion of the fine particle fraction of mechanically-sorted organic fraction of municipal solid waste in laboratory and pilot reactor. *Waste Management* **136**, 83-92 (2021).

11 Liu, C. M. *et al.* Evaluation of methane yield using acidogenic effluent of NaOH pretreated corn stover in anaerobic digestion. *Renewable Energy* **116**, 224-233, doi:https://doi.org/10.1016/j.renene.2017.07.001 (2018).

12 Mustafa, A. M., Li, H., Radwan, A. A., Sheng, K. & Chen, X. Effect of hydrothermal and Ca(OH)2 pretreatments on anaerobic digestion of sugarcane bagasse for biogas production. *Bioresource Technology* **259**, 54-60, doi:https://doi.org/10.1016/j.biortech.2018.03.028 (2018).

13 Cao, W., Sun, C., Li, X., Qiu, J. & Liu, R. Methane production enhancement from products of alkaline hydrogen peroxide pretreated sweet sorghum bagasse. *RSC Advances* **7**, 5701-5707, doi:10.1039/C6RA25798D (2017).

14 Yao, Y., Bergeron, A. D. & Davaritouchaee, M. Methane recovery from anaerobic digestion of urea-pretreated wheat straw. *Renewable Energy* **115**, 139-148, doi:https://doi.org/10.1016/j.renene.2017.08.038 (2018).

15 Bi, S. *et al.* Metabolic performance of anaerobic digestion of chicken manure under wet, high solid, and dry conditions. *Bioresource Technology* **296**, 122342, doi:https://doi.org/10.1016/j.biortech.2019.122342 (2020).

16 Hu, Y.-y. *et al.* Study of an enhanced dry anaerobic digestion of swine manure: Performance and microbial community property. *Bioresource Technology* **282**, 353-360, doi:https://doi.org/10.1016/j.biortech.2019.03.014 (2019).
17 Alamgir, M. & Ahsan, A. Characterization of MSW and nutrient contents of organic component in Bangladesh. *Electronic Journal of Environmental, Agricultural and Food Chemistry* **6**, 1945-1956 (2007).
18 Hossain, H. Z., Hossain, Q. H., Monir, M. M. U. & Ahmed, M. T. Municipal solid waste (MSW) as a source of renewable energy in Bangladesh: Revisited. *Renewable and Sustainable Energy Reviews* **39**, 35-41 (2014).
19 Alamgir, M. & Ahsan, A. Municipal solid waste and recovery potential: Bangladesh perspective. *Journal of Environmental Health Science & Engineering* **4**, 67-76 (2007).
20 Iqbal, S. A. Municipal solid waste management in Sylhet City, Bangladesh. (2017).
